# Evaluation of Sample Preparation Methods for Non-Target Screening of Organic Micropollutants in Urban Waters Using High-Resolution Mass Spectrometry

**DOI:** 10.3390/molecules26237064

**Published:** 2021-11-23

**Authors:** Nina Huynh, Emilie Caupos, Caroline Soares Peirera, Julien Le Roux, Adèle Bressy, Régis Moilleron

**Affiliations:** 1 LEESU, Université Paris-Est Créteil, F-94010 Creteil, France; tinh-nghi-nina.huynh@u-pec.fr (N.H.); emilie.caupos@u-pec.fr (E.C.); soarespereiracaroline@gmail.com (C.S.P.); adele.bressy@enpc.fr (A.B.); moilleron@u-pec.fr (R.M.); 2 LEESU, Ecole des Ponts, F-77455 Marne-la-Vallee, France; 3 OSU-EFLUVE, Université Paris-Est Créteil, F-94010 Creteil, France

**Keywords:** emerging contaminants, high-resolution mass spectrometry, micropollutant fingerprint, non-target screening, solid phase extraction, statistical analysis, urban waters

## Abstract

Non-target screening (NTS) has gained interest in recent years for environmental monitoring purposes because it enables the analysis of a large number of pollutants without predefined lists of molecules. However, sample preparation methods are diverse, and few have been systematically compared in terms of the amount and relevance of the information obtained by subsequent NTS analysis. The goal of this work was to compare a large number of sample extraction methods for the unknown screening of urban waters. Various phases were tested for the solid-phase extraction of micropollutants from these waters. The evaluation of the different phases was assessed by statistical analysis based on the number of detected molecules, their range, and physicochemical properties (molecular weight, standard recoveries, polarity, and optical properties). Though each cartridge provided its own advantages, a multilayer cartridge combining several phases gathered more information in one single extraction by benefiting from the specificity of each one of its layers.

## 1. Introduction

The presence of organic micropollutants in the aquatic environment is a major issue in many countries due to their potential ecotoxicological impact on aquatic organisms as well as their potential risks for human health. European directives concerning the quality of surface waters advocate better control of the presence of micropollutants, but they deal with priority organic molecules that are well known by scientists for their high environmental impact [1]. However, a lot of emerging molecules (e.g., substitutes of forbidden molecules, metabolites, and degradation byproducts) enter the environment due to human activities through wastewater effluents. The survey of these unknown molecules using non-target screening (NTS) enables us to anticipate further regulation on water quality [2]. This analytical approach has undergone high development in the last decade thanks to the spreading of high-resolution mass spectrometry, but it requires powerful analytical systems and computational software as well as non-specific sample preparation methods [3].

The increased resolving power of high-resolution mass spectrometry such as time-of-flight mass spectrometry (TOF-MS) or Orbitrap-MS allows the accurate mass measurement and isotopic pattern identification [4]. Therefore, these techniques are suitable for the development of three main screening strategies based on accurate-mass databases [5,6]: multi-residue target screening [7], suspect screening [8,9,10], and NTS [11,12]. Contrary to target and suspect screening which aim to evaluate the presence of molecules based on a preliminary list, NTS is the characterization of substances present in a sample without any prior information, based on full scan analysis that enables the acquisition of all peaks comprised within a range of masses. It thus requires the subsequent selection (e.g., by statistical analysis) of features of interest (i.e., specific signal characterized by its retention time and *m*/*z* ratio) followed by their tentative identification with the use of databases and literature information [13,14].

In combination with high-resolution mass spectrometry acquisition methods, the computational technique for data processing is of primary interest. Molecular identification depends on high-resolution data precision [13] but also on the software possibilities to deconvolute, filter, re-align signals [15], associate raw formulas to features of interest and finally compare with existing spectra libraries for identification [16,17]. NTS studies can be performed using proprietary software (e.g., Agilent Masshunter and MPP, Waters Unifi, Thermo Compound Discoverer, ABSCiex MarkerView, SpectralWorks AnalyzerPro, associated with ChemSpider, SciFinder, METLIN, PubChem, or MassBank libraries) as well as free ones (e.g., MZmine, R, EnviMass, and MetFrag) [18] for the identification of molecules, using the information on the fragments. The qualification of a single sample requires a lot of computational treatment, and statistical studies are relevant as a complementary approach to assess organic contamination by comparing different samples. Statistical approaches in NTS have been used to differentiate samples in time [19] and space [20] or to highlight variations during processes [11]. Binary comparison, principal component analysis (PCA), and Venn diagrams are examples of methods that can be applied to a sample set in order to isolate features of interest (e.g., molecules in common between samples or specific to groups of samples) [21]. Discriminated samples can be further characterized, and molecules of interest can then be identified.

Data analysis and statistical evaluation of samples being time-consuming, the quality of acquired data must be guaranteed. Sample preparation is essential to ensure the detection of as many molecules as possible and thus needs to be as unselective as possible. Different strategies and methods can be used in order to reach this objective and also to prevent samples from contamination. Direct injection of water samples can be employed to avoid potential contaminations or losses during sample preparation. However, the low concentration of molecules and interferences with other constituents of the sample (e.g., solid particles, colloids, or organic matter) can prevent the detection of trace organic compounds. Preparation techniques such as filtration [22], concentration [23], and solid-phase extraction [24] are widely used for targeted quantitative analysis and for NTS to decrease interferences and to concentrate the samples.

Most NTS studies employ a sample preparation step that is considered to be as “exhaustive” as possible, and little information is available about the efficiency of SPE for NTS purposes. For target screening purposes, knowing the properties of the targeted molecules makes it easier to select the most appropriate cartridge and phase. Concerning NTS studies, the choice of a cartridge is trickier given that, for example, the choice of a cartridge that is recommended for polar compounds will only retain polar compounds and thus, will leave the non-polar compounds un-retained. As a result, most non-target studies employ *universal* phases such as Oasis HLB to retain as many compounds as possible. Validating the extraction and analysis steps is also a challenge for NTS. The choice of standards to assess cartridge efficiency or to evaluate and correct matrix effects are tricky since the compounds studied in NTS are supposed to be unknown. Therefore, a mix of internal standards composed of a large variety of molecules (in terms of molecular weight, polarity, acidity, functional groups, etc.) is often used to assess the efficiency of the method in recovering and analyzing a large panel of compounds [25,26,27,28]. In addition to the injection of internal standards, various methods can be used to check the stability of the instrument or to correct the standard deviation of each feature, such as the injection of a pooled sample (consisting of a mix of all samples to be analyzed), regularly during the sequence. These normalization methods were recently reviewed for NTS applications in lipidomics [29]. Finally, using replicated injections to discard signals that are not repeatable and that could lead to false-positive identifications is a commonly used step in NTS studies [25,28,30,31,32]. Still, only a few studies compared the efficiency of different preparation methods for NTS purposes. One study showed that non-target strategies clearly discriminated the signals in terms of number and type of features obtained with several preparation methods (liquid-liquid extraction and solid-phase extraction on two cartridges) while the extraction yields of targeted molecules were not significantly different between those techniques [33]. In all cases, the properties of the cartridge, the pH of the sample, as well as elution solvents, and conditioning solvents will play a major role in the selection of molecules finally detected in the analyzed sample. A literature review revealed that numerous phases (e.g., HLB, ENV+, C18, X-CW, X-AW, X-C, and X-A, individually or in series), different sample pH, and different conditions of elution were used in NTS studies for environmental applications (Table 1). Several studies used a multilayer cartridge (composed of HLB, ENV+, X-AW, and X-CW) developed to extract a wide range of micropollutants [27].

The overall objective of this work was to assess the influence of the extraction method, in particular the type of SPE phase/cartridge, on the non-targeted HRMS analysis of organic contaminants in urban water. To reach this objective, we pursued the following specific aims: (i) to test different nature of phases to extract a large range of micropollutants in urban waters, based on a panel of 9 commercially-available stationary phases and designed for a variety of applications (i.e., from non-polar/moderately polar to more polar compounds and ionic species); (ii) to develop a strategy based on relevant indicators to compare the results, such as the number of detected molecules, their range, and characteristics; and (iii) to apply the optimized SPE method and the developed data analysis strategy to urban water samples.

## 2. Results and Discussion

### 2.1. Comparison of the Cartridge Retention Ability

In this section, the retention abilities of several cartridges (ENV+, X-A, X-AW, X-CW, HLB pH 2, HLB pH 6, Multilayer, X-C, C18 ENV+, SDBL, and C18) were compared on the basis of different indicators.

#### 2.1.1. Discrimination of Cartridges Based on Optical Properties

**Figure 1:**
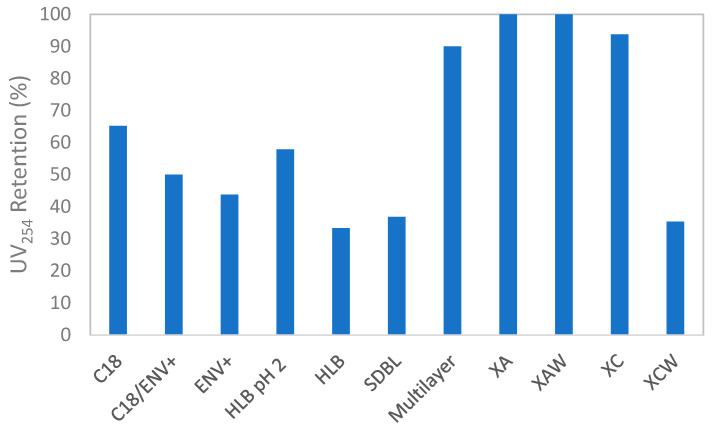
Retention of DOM based on UV absorbance at 254 nm (UV_254_) for different SPE cartridges. UV_254_ retention (%) was calculated as the percentage change between UV_254_ of the Marne River sample and UV_254_ measured at the outlet of each cartridge (during the loading phase).

To get a preliminary overview of the diversity of retained organic materials, optical properties (UV absorbance at 254 nm and 3D-spectrofluorescence spectra) of one sample from a river located in an urbanized area (Marne River) were measured before and after extraction (i.e., on the water phase during the SPE loading step). These optical properties are commonly used to characterize dissolved organic matter (DOM). As DOM is composed of molecules of various sizes and polarities with which micropollutants can interact, the behavior of DOM can be related to the behavior of micropollutants. Based on these interactions (e.g., hydrogen bonding, hydrophobic, van der Waals, or dipole-dipole interactions), fluorescence can be used for a better understanding of the fate of micropollutants, for example, in advanced wastewater treatment processes such as adsorption onto activated carbon [40]. DOM retention on SPE cartridges was evaluated to characterize their ability to retain a large variety of organic materials but also to determine if it can hinder the detection of micropollutants. DOM is indeed known to sometimes reduce the adsorption of target compounds on SPE cartridges (competition effect) and to limit the ionization of molecules for their detection in mass spectrometry [41,42]. The good retention of DOM on SPE cartridges could thus be detrimental to the detection of organic molecules in non-target analyses. Figure 1 presents the retention of DOM on each SPE cartridge based on the percentage change of UV absorbance at 254 nm before and after extraction. The highest recoveries were obtained for X-A, X-AW, X-C, and the Multilayer phases (recoveries ≥ 90%). Other cartridges exhibited significantly lower retention of organic materials (<60%). Especially, extractions with the HLB cartridge retained fewer organic materials (~60%). SDBL, X-CW, and HLB (pH 6) do not seem to be appropriate phases for a large screening of organic material.

For a better evaluation of the quality of DOM retained on the cartridges, 3D-fluorescence spectra were acquired from the Marne River sample before and after extractions. Fluorescence regional integration (FRI) was performed [40,43], calculating the regional fluorescence intensities for each excitation-emission matrix (EEM) spectrum. The EEM spectrum of the Marne River sample before extraction (Supplementary Materials Figure S1) exhibited mainly two fluorophores: one in the region III of FRI and the other in region V. They correspond to large molecules like polysaccharides and humic-like substances [23,44,45]. Other fluorophores (regions I and II) were also observed, representing more hydrophilic and smaller molecules, proteins, and aromatic amino acids [46]. Table 2 presents fluorophores retention depending on the phase of SPE used. Similar results were obtained with other indexes derived from [43] (data not shown).

As expected, the cartridges retained aromatic proteins and humic-like materials (regions I, III, and V [37,40]). Higher retention was observed for XA and XAW (≥85%). Like the results obtained with UV absorbance, the extractions on SDBL and XCW phases led to poor DOM retention (retentions generally lower than 50%). It is interesting to note that regions II and IV were well retained by XA, XAW, and Multilayer (≥80%). They can be associated with the retention of simpler, more polar, and nitrogenous compounds [47]. Based on EEM spectra, these three cartridges seem to be the most efficient to retain hydrophobic materials as well as smaller and more polar compounds for global screening of the molecules present in the sample.

#### 2.1.2. Discrimination of Cartridges Based on HRMS Features

**Figure 2:**
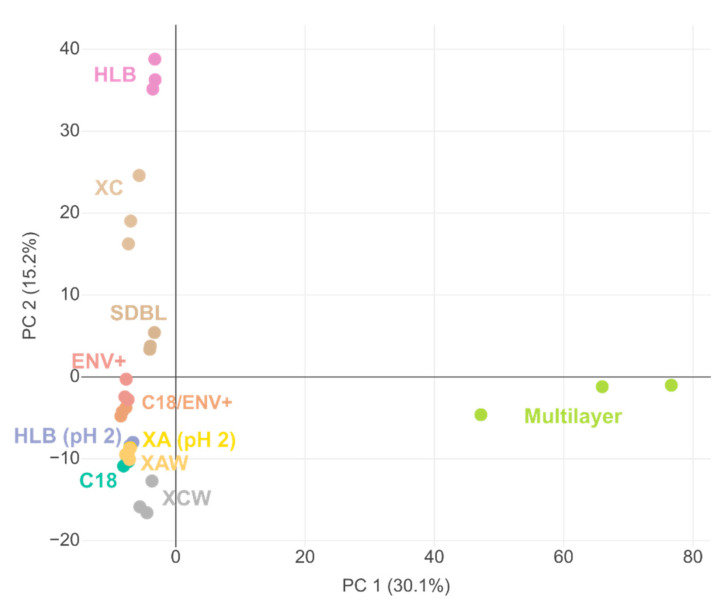
PCA (graph of individuals) of Marne sample extracts on different SPE cartridges.

Samples were then analyzed by HRMS, and differences between SPE cartridges were investigated through criteria classically used in omics studies. A PCA of the HRMS features found in the different extracts of the Marne River sample was performed to quickly identify similarities and differences between the feature sets obtained from each cartridge (Figure 2).

All cartridges were clearly discriminated by the first two components of the PCA (covering 45.3% of the total variance), which indicated that each type of SPE phase extracted specific sets of HRMS features. The first component (PC 1) was mostly correlated with the features derived from the Multilayer cartridge and explained 30% of the dataset variability. The second component (PC 2) explained 15% of the variability and discriminated all individual cartridges. Polymeric cartridges (ENV+, SDBL, and C18/ENV+) were all clustered near the center of the PCA, meaning that they all extracted similar features. Cartridges for which sample loading was performed at pH 2 (HLB pH 2 and XA) also exhibited similar behavior. Features from anionic exchange cartridges (XA and XAW) were almost identical.

**Figure 3:**
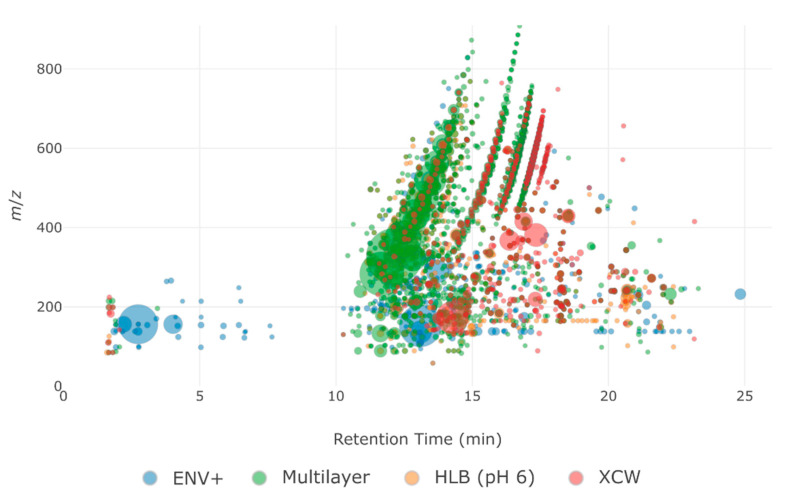
Fingerprints of detected features from the Marne River sample after SPE on ENV+ (blue), HLB (orange), Multilayer (green), and XCW (red) cartridges. The size of bubbles is proportional to the area of the feature.

To better understand the repartition of cartridges obtained by the PCA (Figure 2), fingerprints (i.e., bubble plots of all detected features) were compared between the most different cartridges (XCW, ENV+, HLB, and the Multilayer cartridge) for the Marne river sample (Figure 3).

**Figure 4:**
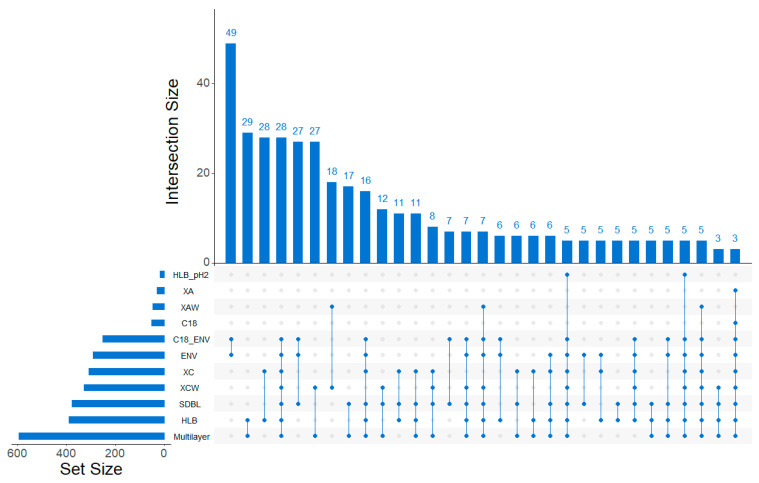
Upset diagram, representing the intersection of the different cartridges in terms of retained features. The “set size” represents the total number of features from each cartridge, the “Intersection size” represents the size of the intersection (i.e., number of features in the intersection designated by the dots), the dots represent the cartridges intersected (i.e., number of intersections).

The Multilayer cartridge displayed the most intense features on the entire *m*/*z* range, whereas ENV+ displayed more intense features for *m*/*z* < 400, and HLB retained more intense features with *m*/*z* > 400. XCW showed more intense signals at retention times greater than 15 min (i.e., less polar compounds), whereas most intense signals on the two other individual cartridges were obtained at retention times before 15 min. Thus, the PC 2 component of the PCA may describe the polarity of the retained features on each cartridge. This hypothesis is supported by the fact that the C18 cartridge, which is designed for less polar compounds, was close to the XCW one on the PCA. Finally, a comparison of common features retained by the cartridges was performed (Figure 4).

The first thing to notice is that the Multilayer cartridge had the biggest set size among the studied cartridges, meaning that it retained the highest number of features. The two closest cartridges in terms of retained features were the ENV+ and C18/ENV+ cartridges with 49 common features, which is not surprising given the similar nature of the phases. The cartridge involved in the highest number of intersections was the SDBL (23 intersections for 210 features intersected); however, the cartridge with the highest number of features intersected was the Multilayer one (20 intersections for 212 features intersected). Therefore, the Multilayer was the cartridge with the highest number of specific features (323 features only found in this cartridge), but also the one with the highest number of intersected features, making it the most interesting cartridge for non-target studies by retaining the most extensive set of features.

Compared to the results obtained when considering the optical properties (Section 2.1.1), the cartridges that retained more fluorescent organic materials exhibited a lower number of HRMS features (e.g., XA, XAW). On the contrary, SDBL and XCW showed low retention of fluorescing materials but a high number of HRMS features. This could be due to the competition of DOM with organic compounds for the adsorption on the SPE cartridge [41,48] or to matrix effects in the HRMS analysis. Interestingly, this behavior can also be observed for the same cartridge used at different pH. Extraction on HLB at pH 6 gave a high number of features in HRMS and low retention of fluorescing organic materials (fluorophores), contrary to the extraction on HLB at pH 2. Notably, the Multilayer cartridge exhibited both retention of fluorophores and a large number of HRMS features; this cartridge thus seemed less affected by organic content.

### 2.2. Characterization of the Cartridge Retention Ability with Different Matrices

Based on the previous observations (HRMS fingerprints and DOM retention), five cartridges were retained for further investigation with different matrices: the Multilayer cartridge (highest number of retained features and highest intensities), HLB at pH 6 (universal cartridge, used in many studies), ENV+ (universal cartridge, representing the polymeric cartridges clustered in the PCA), XAW (high retention of fluorophores) and XCW (retaining more apolar compounds). Three types of samples were extracted and compared, with the increasing complexity of matrix (i.e., presence of organic and inorganic constituents): Ultrapure water (Milli-Q), surface water (Marne River), and wastewater effluent (WWe).

#### 2.2.1. Recoveries of Internal Standards

**Figure 5:**
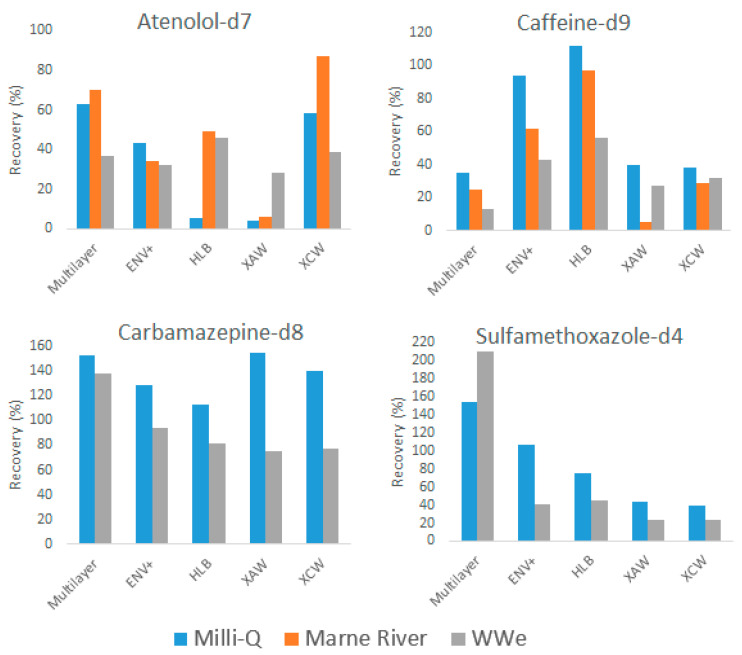
Recovery of internal standards after extraction by the cartridges for different matrices.

To compare the different SPE cartridges and evaluate the corresponding matrix effects, the recovery of internal standards was first calculated (Figure 5).

In Milli-Q, recoveries ranged from 4% to 63% for atenolol-d7, from 35% to 112% for caffeine-d9, from 113% to 154% for carbamazepine-d8 and from 40% to 155% for sulfamethoxazole-d4. Carbamazepine-d8 exhibited an overall great recovery with all cartridges and showed the least differences between cartridges. On the contrary, atenolol-d7 gave lower recoveries, and sulfamethoxazole-d4 and caffeine-d9 exhibited large differences in recoveries between cartridges. Carbamazepine-d8 has the highest log Kow among the four molecules; its better recovery on all cartridges could be explained by its lower hydrophilicity. Extractions were all carried out at pH = 6.5–7, meaning that, for atenolol-d7, caffeine-d9 and carbamazepine-d8, the acidic form was predominant, whereas, for sulfamethoxazole-d4, its basic form is predominant. The only molecule showing a significant difference between XAW and XCW cartridges (designed for compounds with pKa < 5 and pKa > 8, respectively) was atenolol-d7 (pKa = 9.6), with a better recovery on XCW as expected. The strong acidity (pKa of sulfamethoxazole-d4 = 1.6) or basicity (pKa of caffeine-d9 and carbamazepine-d8 are 14 and 13.9, respectively) of the three other compounds may explain their less contrasted retention of those two cartridges. Overall, the Multilayer cartridge gave the highest recoveries of those standards (despite a quite poor recovery of caffeine-d9).

Lower recoveries of caffeine-d9 were recorded with the Marne River and WWe samples for all five cartridges as compared to the Milli-Q water sample. The three universal cartridges (Multilayer, HLB, and ENV+) were more affected by complex matrices. The ionic XCW cartridge showed the least difference in recoveries for the three matrices. However, the XAW cartridge exhibited a very low recovery of most internal standards from the Marne River sample, while the two other samples (Milli-Q and WWe) showed similar recoveries. Better retention of caffeine-d9 was expected on the XCW cartridge because of its high pKa value of 14. Compared to other cartridges, recoveries of caffeine-d9 on XCW was low but more consistent across the different types of water. Its lower recovery can be explained by the employed elution step that was recommended by the cartridge supplier for specific compounds (Alprenolol, Acetaminophen, and Clomipramine). The elution step could be optimized with this cartridge to obtain better recoveries for internal standards such as caffeine-d9 as well as for the total number of features retained.

An unexpected high signal was obtained for carbamazepine-d8 and sulfamethoxazole-d4 in the Marne River sample after SPE, leading to excessive recovery values (>1000%), which were therefore not included in Figure 5. The extraction of those two components on WWe, led to signal suppression as compared to the Milli-Q sample. Such matrix effects were previously reported in wastewater effluent with ion suppression of 34% for sulfamethoxazole, 5.4% for caffeine, and 23% for carbamazepine on Strata-X cartridges [49]. Atenolol-d7 exhibited various behaviors, with a signal enhancement for HLB and XAW, signal suppression for ENV+, and both signal suppression (WWe sample) and signal enhancement (Marne River sample) for the Multilayer cartridge and XCW.

Matrix effects (i.e., signal suppression or enhancement) or adsorption competition on the cartridge were observed for all detected internal standards. A high suppression was especially observed for the XAW cartridge, in accordance with the impact of adsorbed organic materials already described in Section 2.1.2.

#### 2.2.2. Range and Properties of Retained Features

The number of features retained on each cartridge was compared for the WWe and the Marne River sample (Table 3). As mentioned previously, the Multilayer cartridge displayed the highest number of features compared to the other cartridges, which explains its widespread use in non-target screening studies [27,35,36]. HLB and ENV+, commonly considered as “universal cartridges”, were also quite efficient for the Marne River sample, retaining 32% and 47% fewer features than the Multilayer cartridge, respectively. They also retained the highest number of features from the WWe sample, ENV+ being the most efficient. XCW retained 11% more features from the Marne River sample than ENV+, which suggests the presence of cationic substances in this sample. Table 3 displays the properties of the features retained on each cartridge for both samples. 

The Multilayer cartridge retained globally bigger molecules than other cartridges, as seen from both average *m*/*z* and weighted average *m*/*z*. For the two types of samples, HLB also retained features with larger molecular sizes and ENV+ smaller ones. When those values were not weighted by the feature area, no clear trend could be observed. The polarity of retained features (as described by the average retention) was also not significantly different between the cartridges. However, it can be noted that the Multilayer cartridge average retention of features from the Marne River sample (46% ACN) was equal to the mean of the average retention of all four other cartridges (i.e., the four phases used in the Multilayer cartridge). For the WWe sample, the total area of the detected features followed the same ranking order as the total number of features (i.e., ENV > HLB > XAW > XCW). However, this was not the case for the Marne River sample, for which ENV+ exhibited 15% less detected features compared to HLB but a slightly more intense total signal. This observation indicates that the molecules retained on ENV+ were more ionizable, therefore different from HLB, or better retained, as it was demonstrated by the difference in their fingerprints (Figure 3). The Multilayer cartridge showed the highest total intensity among all cartridges, which demonstrates its ability to retain a large number of compounds with a high signal. This property is clearly beneficial for NTS studies to obtain clean mass spectra and thus to allow easier identifications.

**Figure 6:**
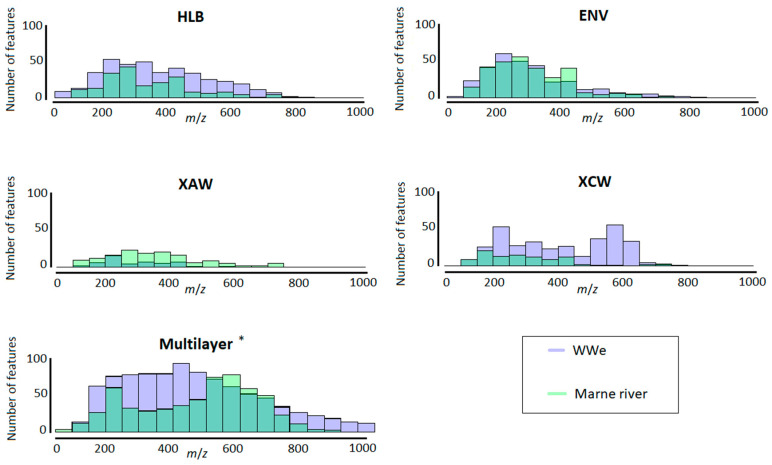
*m*/*z* distribution of the retained features on the different cartridges. * Number of features for Multilayer WWe were divided by eight.

The distribution of *m*/*z* values was visualized for both types of the sample matrix and for each cartridge (Figure 6). To the authors’ knowledge, this representation has not yet been used for the characterization of HRMS data, although it is useful for identifying differences between samples. Comparison of the cartridges shows that ENV+ was less effective in retaining molecules with *m*/*z* > 600, whereas HLB covered a larger range of *m*/*z*. It is interesting to note that the Multilayer cartridge combined the ranges of *m*/*z* distribution of ENV+, HLB, XAW, and XCW.

### 2.3. Application: Evaluating a Disinfection Treatment by Performic Acid

**Figure 7:**
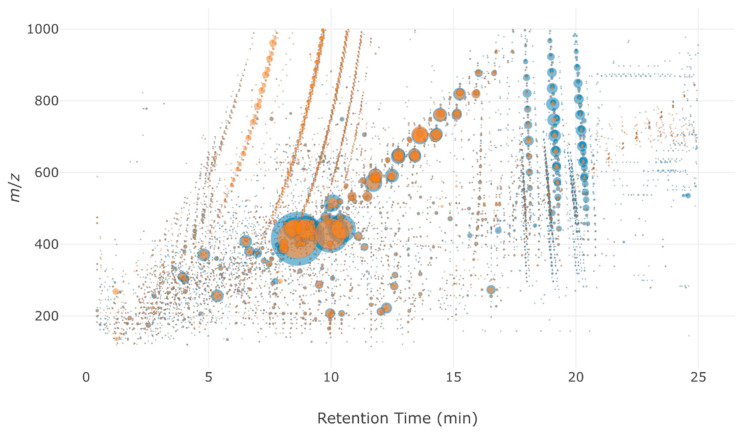
Bubble plot of WWe (blue) and WWe after 30 ppm PFA treatment during 10 min (orange) extracted on a Multilayer cartridge.

The above-mentioned indicators showed that the Multilayer cartridge was the most efficient among the different tested cartridges and that it is the most suitable to be used as a universal cartridge. It was therefore tested in a real-case application to characterize the evolution of organic compounds during a disinfection treatment of wastewater effluents by performic acid (PFA). Raw wastewater effluent and the same effluent treated with 30 ppm of PFA (contact time of 10 min) were extracted with the Multilayer cartridge and analyzed by HRMS (Figure 7).

The Multilayer cartridge allowed the detection of a wide number of compounds covering the whole range of *m*/*z* values at low and high retention times. Sample after treatment showed a significant decrease in the area of the markers with a retention time between 17 and 21 min and an increase for the markers between 5 and 7 min compared to the non-treated wastewater.

This observation suggests that compounds with lower polarity (i.e., higher retention time) were transformed into more polar ones by the treatment. Table 4 displays the distribution of the number of markers before and after treatment in different zones defined by the *m*/*z* and retention time ranges. Zone 2 (bigger and more polar molecules) exhibited a significant increase in both the number of markers and their total area. Zone 1 and zone 3 (molecules with *m*/*z* < 500) showed a decrease by almost a third of the number of markers after treatment, and a decrease in their total intensity as well. Finally, PFA treatment slightly increased the number of markers in zone 4 (bigger and less polar molecules), but the overall intensity of signals decreased in that zone. Globally, these results show that PFA treatment formed more polar molecules and removed a larger proportion of small molecules.

## 3. Materials and Methods

### 3.1. Chemicals and Standards

All standards were obtained from Sigma-Aldrich (Dr. Ehrenstorfer, Augsburg, Germany, purity > 99%). Methanol (MeOH), ammonia (35%), hydrochloric acid (37%), and formic acid (98%) were LCMS grade and purchased from Fischer Scientific (Illkirch Cedex, France). Ethyl acetate (EtAc), dichloromethane (DCM), and acetone were obtained from Merck (Darmstadt, Germany), and acetonitrile (ACN) was purchased from Biosolve (Dieuze, France), at analytical grade. HPLC water (Milli-Q) was produced from deionized water using a Millipore Milli-Q system (IQ 7000, Merck, Darmstadt, Germany) equipped with an LC-pak polisher (Merck, Darmstadt, Germany).

Deuterated standards were used to evaluate matrix effects from different samples, to evaluate the extraction efficiency on the various SPE phases, and to correct the retention time of the chromatograms. A mixed solution of 4 deuterated compounds (atenolol-d7, caffeine-d9, carbamazepine-d8, sulfamethoxazole-d4) was prepared in MeOH at a concentration of 10 mg/L for further injection in samples.

Before sampling and analysis, glassware was washed with TFD4 (Franklab, Montigny-le-Bretonneux, France), rinsed with deionized water, and calcined at 500 °C to remove any trace of organic contamination.

### 3.2. Sample Collection and Preliminary Preparation

Various types of samples were used: Milli-Q water produced in the laboratory, surface water under anthropic pressure (Marne river, France), and wastewater effluent (WWe).

Thirty liters of water from the Marne River were collected from a bridge at Chennevières-sur-Marne (GPS coordinates: 48.79071188130754, 2.5215935793518462) downstream the wastewater treatment plant of *Marne Aval* (Noisy-le-Grand, France). The sample was collected in 3 × 10 L amber glass bottles and filtered the same day, using 0.7 µm glass fiber filters (GF/F Whatman). After homogenization, aliquots of 500 mL were prepared and completed to 1 L with Milli-Q water in order to avoid potential matrix effects occurring during the SPE loading step. The initial pH and dissolved organic carbon (DOC) were 8.5 and 1.89 mg C/L, respectively. Some samples were acidified, either to pH 6.5 with 50 µL of formic acid or to pH 2–3 with 400 µL of formic acid according to recommendations for specific SPE cartridges.

Ten liters of treated effluent were collected in June 2019 from the wastewater treatment plant of *Seine Amont* (Valenton, France) in 10 L amber glass bottles and filtered on 0.7 µm glass fiber filters (GF/F Whatman). The initial pH and DOC were 7.9 and 7.1 mg C/L, respectively. One liter subsamples were acidified to pH 6.5 with 50 µL of formic acid.

### 3.3. SPE Cartridges

#### 3.3.1. Selection of Cartridges

Strata-X cartridges (500 mg, 6 mL) (Phenomenex, Le Pecq, France) are ionic-based polymeric sorbents, chosen to retain compounds according to their pKa: Strata X-AW (for pKa < 5), Strata X-CW (for pKa > 8), Strata X-A (for pKa < 2), Strata X-C (for pKa > 10.5). Oasis HLB (200 mg, 6 mL) (Waters, Milford, MA, USA) is a reversed-phase cartridge used to select a wide range of substances, including neutral, basic, acidic, and polar ones. Silica-based cartridges Strata C18 (200 mg, 6 mL) and Strata SDBL (500 mg, 6 mL) (Phenomenex, Le Pecq, France) are employed to retain neutral hydrophobic and non-polar molecules. ENV+ (500 mg, 6 mL) (Biotage, Glamorgen, UK) is a polymeric phase appropriate for the retention of polar analytes. C18/ENV+ (400 mg, 6 mL) (Biotage, Glamorgen, UK) is a layered cartridge composed of the previous ENV+ phase as the bottom layer and a C18 phase as the top layer to improve the range of analytes possibly extractable. Finally, a homemade Multilayer cartridge made of Oasis HLB (200 mg), Isolute ENV+ (150 mg), Strata X-AW (100 mg), and Strata X-CW (100 mg) was prepared according to the method developed by Kern et al. [27].

#### 3.3.2. Extraction Protocol

Samples were spiked with the mix of internal standards at 100 ng/L, 24 h prior to extraction and stored at 4 °C. Experimental conditions for conditioning, loading, and elution on each cartridge followed recommendations of suppliers, or the method developed by Kern et al. for the Multilayer cartridge [27], and are described in Table 5. All extractions were carried out on Visiprep (Sigma-Aldrich, Augsburg, Germany) and Autotrace (AT280, Caliper) SPE systems. All cartridges were loaded with 1 L of sample at pH 6–7, except for Strata X-A (pH 2–3 recommended) and Oasis HLB, for which both pHs were tested. Before elution, the cartridges were dried for 30 min. After elution, the extracts were stored in the dark at 4 °C prior to their analysis. SPE extracts were evaporated under a stream of nitrogen, then reconstituted in 1 mL of Milli-Q water and MeOH (80/20, *v*/*v*), and filtered through 0.2 µm PTFE filters before a 10 µL injection on the analytical system. 

### 3.4. UV-Visible and 3D Fluorescence Analyses

Spectroscopic measurements were performed on samples before and after filtration on the SPE cartridges (loading phase). UV-Visible analyses were conducted on a spectrophotometer (UviLine 9400, Secomam, Aqualabo, Champigny-sur-Marne, France), and 3D-fluorescence spectra were obtained by a spectrofluorometer (FP-8300, Jasco, Pfungstadt, Germany). Those apparatuses were both equipped with a Xenon lamp and a 1 cm-quartz cell. For the fluorescence spectra, EEM were generated by scanning excitation wavelengths from 240 to 450 nm (every 5 nm), and the emission wavelengths were detected between 250 to 600 nm (every 2 nm), at a scan speed of 1000 nm/min and response at 0.1 s. To avoid any effect of the intern filter, samples with an absorbance at 254 nm higher than 0.1 were diluted.

3D-fluoresence indexes where chose according to previous studies [40,43]. Fluorescence regional integration (FRI) was performed, regions I to V were calculated and compared.

### 3.5. LC-HRMS Analyses

LC-HRMS analyses of the Marne River samples were conducted on SYNAPT HDMS QTOF (Waters) coupled with a Nano ACQUITY UPLC system (Waters). The column was an ACQUITY UPLC Peptide BEH C18, 130 Å (100 µm × 100 mm, 1.7 µm). The mobile phase was Milli-Q water with 0.1% formic acid (A) and ACN (B) following the gradient described in Supplementary Materials Table S1, for a total run time of 40 min. Analyses were performed in positive mode (ESI+) with screening between 50 and 1000 *m*/*z*. LC-HRMS analyses of Milli-Q and WWe samples were performed with a Vion–UPLC-IMS-QTOF (Waters) equipped with an ACQUITY UPLC BEH C18 (2.1 × 100 mm, 1.7 µm) column and the corresponding pre-column. The mobile phase was Milli-Q water + 0.1% formic acid (A) and ACN + 0.1% formic acid (B) following the gradient described in Supplementary Materials Table S1, for a total run time of 34 min. Analyses were performed in positive mode (ESI+) with screening between 50 and 1000 *m*/*z*. To ensure data quality, several steps were implemented.

Quality insurance procedure: Before each analysis, a quality reference standard, consisting of 9 components (Acetaminophen, Caffeine, Leucine enkephalin, Reserpine, Sulfadimethoxine, Sulfaguanidine, Terfenadine, Val-tyr-val, Verapamil), was injected five times to check the system performance by calculating the mass error deviation, the average peak width and, when available, the CCS (collision cross section) error for those compounds. If the mass error deviation was higher than 2 ppm or the peak width was longer than 3.0 sec, a system calibration was conducted. The same procedure was repeated until the expected conditions were met. Each sampling sequence began with three blank injections to wash the column and five pool injections to stabilize the column. The pool sample consists in a mix of equal volume of each sample and is used as a quality control. Each sample was then injected in randomized triplicate to minimize the effect of instrumental deviation. For data treatment, only the features detected in every replicate of a given sample were considered. Every 10 injections, a pool was re-injected to monitor the system. The clustering of these pool samples in the PCA was checked to assess the reproducibility of the system.

Raw HRMS data were exported directly after acquisition and converted in *.mzML format via MSConvert (Version 3) [50,51] for further treatment in R software (Version 3.6.2). Pre-treatment of raw data was performed with the *Patroon* package [52], using OpenMS for peak picking and alignment. Features that were not present in all replicates of a given sample were discarded. PCA was performed with the *FactoMineR* [53] and *factoextra* [54] packages, and Upset diagrams were plotted with the *UpSetR* package [55].

The extraction efficiency of an SPE cartridge was determined based on the area of detected internal standards in the samples compared to the area of a standard mix injected. The number of detected features and the total area of the signal (i.e., sum of the areas of each feature) were compared for each cartridge. The average *m*/*z* and average retention of a sample were calculated from the *m*/*z* values and retention times of each feature detected in the sample. Retention was expressed as the percentage of ACN needed to elute a given feature at the corresponding retention time. Values of average *m*/*z* and average retention were also weighed by the area of each feature.

## 4. Conclusions

The goal of this work was to compare the efficiency of several commercially available SPE cartridges and of a homemade Multilayer cartridge (as developed by Kern et al. [27]) for non-target screening purposes. Various parameters were monitored, such as the recovery of a limited number of analytical standards, the number and properties of detected features in HRMS, or the global retention of DOM (through 3D fluorescence measurements) on each cartridge. The Multilayer cartridge was the most effective at retaining internal standards and a large range of HRMS features, and it was not as affected by the adsorption of DOM as other cartridges (i.e., adsorption competition effects and/or matrix effects). It seemed to take advantage of the four phases involved in its composition (HLB, ENV+, XCW, and XAW) in terms of diversity of compounds (i.e., polarity and molecular size).

Although the other cartridges had some specificity missed by this Multilayer cartridge (e.g., an important number of specific features was only retained on the ENV+ cartridge), its efficiency in extracting a great number of compounds was clearly demonstrated. This observation could be further confirmed by using different analytical tools (e.g., gas chromatography) or different ionization modes and sources to increase the range of molecules detected. Moreover, a further investigation involving the tentative identification of some retained features could be performed. The optimization of extraction conditions (e.g., masses and combinations of sorbents, types, and volumes of eluting solvents) could also be investigated to retain an even greater number of features. It would finally be useful to better characterize the chemical space (log Kow, pKa, chemical functions, etc.) covered by each cartridge. A larger number of internal standards covering a broader range of chemical properties could thus be studied. This could help determine the relevance of each type of cartridge towards specific purposes (e.g., the detection of a larger number of polar or ionic compounds or some specific families of molecules).

## Figures and Tables

**Table 1:** Examples of SPE cartridges, pH of samples, and elution solvents used for sample preparation in environmental studies using non-target screening for the analysis of water samples.

Matrix	pH	SPE Cartridge	Elution Solvents	Ref
Surface water and wastewater (influent and effluent)	N.A. ^a^	HLB	N.A. ^a^	[8]
Surface water, groundwater and Drinking water	2	HLB, MCX	Acetonitrile (HLB) Acetonitrile, Acetonitrile + 5% ammonia (MCX)	[34]
Surface water, wastewater influent and effluent	6.7	Multilayer (HLB, ENV+, X-AW, X-CW)	Methanol/Ethyl acetate (50:50, *v*/*v*) + 2% ammonia and methanol/ Ethyl acetate (50:50, *v*/*v*) + 1.7% formic acid	[11,26,27,35,36,37]
Landfill leachate and groundwater	7 and 3	ENV+	Methanol	[21]
Wastewater effluent	2	MCX and Strata X in series	Methanol + 5% ammonia	[38]
Wastewater (influent and effluent)	N.A. ^a^	HLB	Methanol	[39]
Surface water	N.A. ^a^	Multilayer (HR-X, HR-XAW, HR-XCW)	Ethyl acetate; methanol; methanol + 2% ammonia and methanol + 1% formic acid	[5]
Wastewater effluent	N.A. ^a^	MAX and MCX in series	Methanol/ethyl acetate/formic acid (69:29:2, *v*/*v*) (MAX) methanol/ethyl acetate/ammonia solution (67.5:27.5:5, *v*/*v*) (MCX)	[10]
Riverbank filtration system	N.A. ^a^	HLB	Methanol	[32]

^a^ N.A. = Not Available.

**Table 2:** DOM retention (%) based on fluorophores for different SPE cartridges (indexes FRI derived from [43]).

Index	C18	C18 ENV+	ENV+	HLB pH 2	HLB	Multilayer	SDBL	XA	XAW	XC	XCW
Region I FRI	84	85	64	70	58	71	58	94	89	55	49
Region II FRI	76	69	67	68	58	86	48	96	90	59	47
Region III FRI	72	46	46	55	55	73	27	92	86	50	28
Region IV FRI	68	62	64	65	45	80	41	93	86	53	45
Region V FRI	70	47	50	56	53	55	28	93	86	48	28

**Table 3:** Properties of features retained on various SPE cartridges. The average retention is given as the percentage of acetonitrile in the mobile phase (ACN) needed to elute a given feature. The weighted values correspond to the average parameter, weighted by the intensity of each individual marker.

	Number of Features	Sum of Detected Areas	Average *m*/*z*	Weighted Average *m*/*z*	Average Retention (% ACN)	Weighted Average Retention (% ACN)
	**Marne River**					
ENV	315	4.25 × 10^6^	306.6403	300.0973	43	41
HLB	403	4.20 × 10^6^	367.9942	379.1581	44	43
XAW	49	8.69 × 10^5^	295.4186	323.9237	52	52
XCW	350	3.43 × 10^6^	404.9278	366.7695	48	46
Multilayer	594	5.84 × 10^6^	470.6467	441.9003	46	44
	**WWe**					
ENV	301	2.72 × 10^7^	306.7059	339.2997	37	33
HLB	201	1.75 × 10^7^	337.8578	311.8444	35	27
XAW	149	9.03 × 10^7^	345.6214	384.1570	34	22
XCW	100	8.32 × 10^6^	297.6594	367.0206	28	23
Multilayer	7515	4.89 × 10^7^	461.3586	416.1775	47	35

**Table 4:** Distribution of markers before and after PFA treatment.

	Before PFA		After 30 ppm PFA	
	Number of Markers	Sum of Detected Areas	Number of Markers	Sum of Detected Areas
Zone 1 *m*/*z*: 0–500 Retention Time: 0–10 min	4861	2.51 × 10^8^	3781	1.85 × 10^8^
Zone 2 *m*/*z*: 500–1000 Retention Time: 0–10 min	996	0.35 × 10^8^	2301	1.36 × 10^8^
Zone 3 *m*/*z*: 0–500 Retention Time: 10–25 min	2795	1.43 × 10^8^	1728	0.75 × 10^8^
Zone 4 *m*/*z*: 500–1000 Retention Time: 10–25 min	2770	2.93 × 10^7^	3557	1.95 × 10^8^

**Table 5:** Description of the methods used for water sample extraction on the different SPE cartridges.

Cartridge	Sample	Conditioning	Washing	Eluting
X-A	1 L pH 2–3 Marne	10 mL MeOH 10 mL Milli-Q	10 mL MeOH	10 mL MeOH + 5% formic acid
X-AW	1 L pH 6–7 Marne, MQ, WWe	10 mL MeOH 10 mL Milli-Q	10 mL MeOH	5 mL MeOH + 5% ammonia 5 mL MeOH + 5% formic acid
X-C	1 L pH 6–7 Marne	10 mL MeOH 10 mL Milli-Q pH = 2	10 mL MeOH + 0.1 M HCl	5 mL MeOH + 0.1M HCl 5 mL MeOH + 5% ammonia
X-CW	1 L pH 6–7 Marne, MQ, WWe	10 mL MeOH 10 mL Milli-Q	10 mL MeOH	5 mL MeOH + 5% formic acid 5 mL MeOH + 5% ammonia
HLB	1 L pH 2–3 Marne 1 L pH 6–7 Marne, MQ, WWe	10 mL MeOH 10 mL AcEt 5 mL DCM 10 mL Milli-Q	No washing	5 mL MeOH 5 mL AcEt 5 mL DCM
ENV+	1 L pH 6–7 Marne, MQ, WWe	10 mL MeOH 10 mL Milli-Q	5 mL Milli-Q /MeOH (95/5, *v*/*v*)	5 mL MeOH 5 mL acetone + 5% ammonia
C18	1 L pH 6–7 Marne, MQ, WWe	10 mL MeOH 10 mL Milli-Q	No washing	5 mL MeOH 5 mL acetone + 5% ammonia
C18/ENV+	1 L pH 6–7 Marne	10 mL MeOH 10 mL Milli-Q	No washing	5mL MeOH 5mL acetone + 5% ammonia
SDBL	1 L pH 6–7 Marne	10 mL MeOH 10 mL Milli-Q pH = 4	No washing	5 mL MeOH 5 mL acetone + 5% ammonia
Multilayer	1 L pH 6–7 Marne, MQ, WWe	10 mL MeOH 10 mL Milli-Q	No washing	6 mL AcEt/MeOH (50/50, *v*/*v*) + 1.43% ammonia 3 mL AcEt/MeOH (50/50, *v*/*v*) + 1.7% formic acid

## Data Availability

Raw HRMS data of Marne river samples are available at https://doi.org/10.5281/zenodo.5589621, accessed on 18 November 2021.

## Supplementary Materials

The following are available online. Figure S1: EEM matrices of Marne river (A) before extraction, (B) after extraction on X-A, (C) after extraction on HLB, (D) after extraction on C18/ENV+, (E) after extraction on SDBL, (F) after extraction on the Multilayer cartridge, Table S1: Description of the gradient used for non-target analysis.

## Sample Availability

Samples of the compounds are available from the authors.
