# Supplementary material for: Evaluation of Sample Preparation Methods for Non-Target Screening of Organic Micropollutants in Urban Waters Using High-Resolution Mass Spectrometry
Source: Molecules. 2021 Nov 23;26(23):7064. doi: 10.3390/molecules26237064 (PMC8659043; doi:10.3390/molecules26237064)
Supplement: Supplementary file 1 [file molecules-26-07064-s001.zip › molecules-1469612-supplementary.pdf]

Evaluation of Sample Preparation Methods for Non-Target Screening of Organic Micropollutants in Urban Waters Using High-Resolution Mass Spectrometry

Nina Huynh, Emilie Caupos, Caroline Soares Peirera, Julien Le Roux, Adèle Bressy and Régis Moilleron

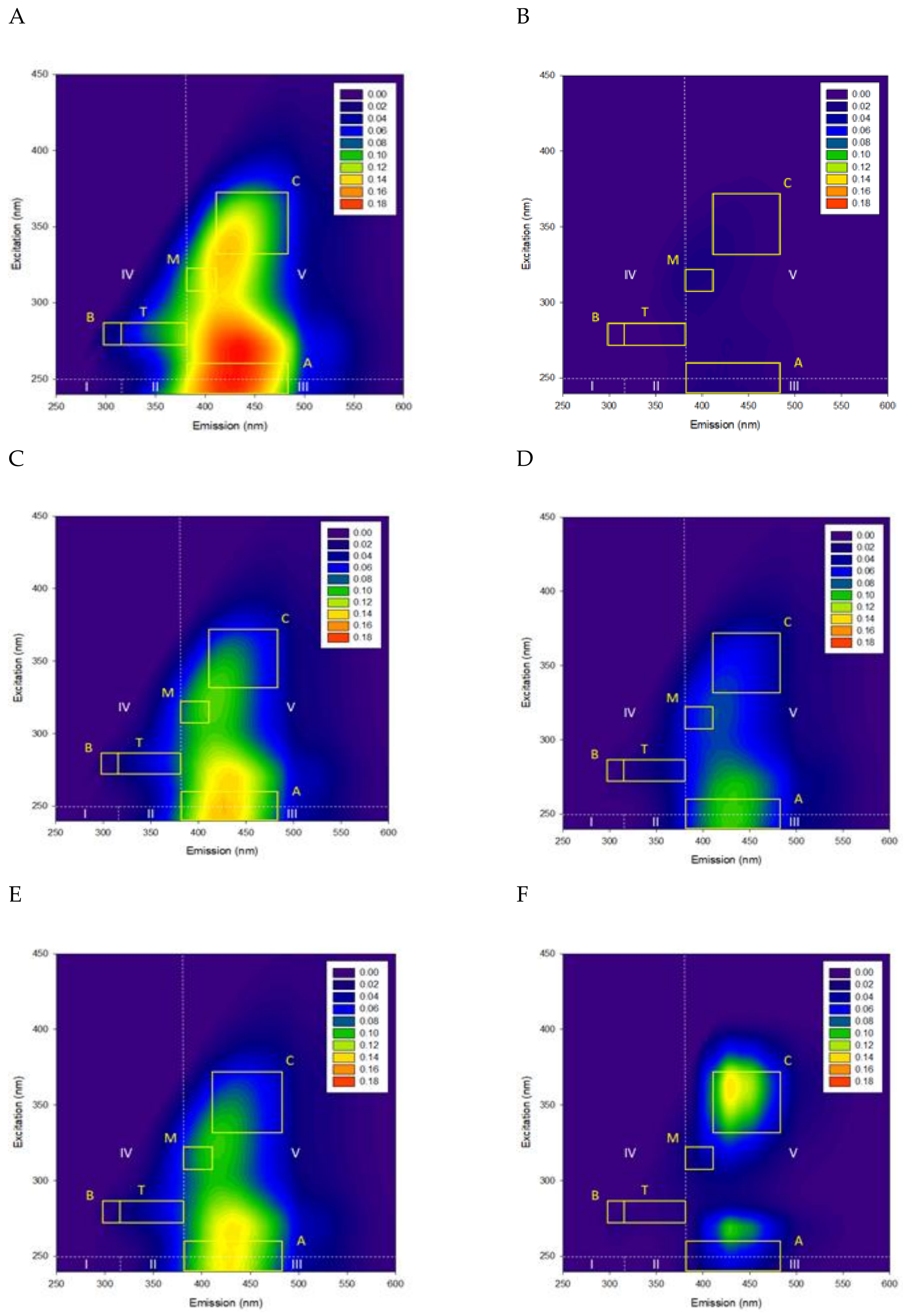

Figure S1: EEM matrices of Marne river (A) before extraction, (B) after extraction on X-A, (C) after extraction on HLB, (D) after extraction on C18/ENV+, (E) after extraction on SDBL, and (F) after extraction on the multilayer cartridge

Table S1: Description of the gradient used for non-target analysis

Nano ACQUITY UPLC gradient			Vion–UPLC-IMS-QTOF gradient		
**Time (min)**	**A (%)**	**B (%)**	**Time (min)**	**A (%)**	**B (%)**
0	99	1	0	98	2
2	99	1	1	98	2
32	0	100	25	2	98
37	0	100	30	2	98
40	99	1	31.5	98	2
45	99	1	34	98	2

Text S1: Quality insurance procedure

Before each analysis, a quality reference standard, consisting of nine components (Acetaminophen, Caffeine, Leucine enkephalin, Reserpine, Sulfadimethoxine, Sulfaguanidine, Terfenadine, Val-tyr-val, and Verapamil), was injected five times to check the system performance by calculating the mass error deviation, the average peak width and, when available, the CCS (collision cross-section) error for those compounds. If the mass error deviation was higher than 2 ppm or the peak width was longer than 3.0 sec, a system calibration was conducted. The same procedure was repeated until the expected conditions were met. Each sampling sequence began with three blank injections to wash the column and five pool injections to stabilize the column. The pooled sample consisted of a mix of an equal volume of each sample and was used as a quality control. Each sample was then injected in randomized triplicate to minimize the effect of instrumental deviation. For data treatment, only the features detected in every replicate of a given sample were considered. Every 10 injections, a pool was re-injected to monitor the system. The clustering of these pool samples in the PCA was checked to assess the reproducibility of the system.
